# Selected Concrete Models Studied Using Willam’s Test

**DOI:** 10.3390/ma13214756

**Published:** 2020-10-24

**Authors:** Adam Wosatko, Michał Szczecina, Andrzej Winnicki

**Affiliations:** 1 Faculty of Civil Engineering, Cracow University of Technology, Warszawska 24, 31-155 Cracow, Poland; andrzej.winnicki@pk.edu.pl; 2 Faculty of Civil Engineering and Architecture, Kielce University of Technology, al. Tysiąclecia Państwa Polskiego 7, 25-314 Kielce, Poland; m.szczecina@tu.kielce.pl

**Keywords:** concrete models, Willam’s test, damage, plasticity, smeared cracking, nonlinear analysis, FEM

## Abstract

Willam’s test is a quick numerical benchmark in tension–shear regime, which can be used to verify inelastic (quasi-brittle) material models at the point level. Its sequence consists of two separate steps: uniaxial tension accompanied with contraction—until the tensile strength is attained; and next for softening (cracking) of the material—tension in two directions together with shear. A rotation of axes of principal strains and principal stresses is provoked in the second stage. That kind of process occurs during the analysis of real concrete structures, so a correct response of the material model at the point level is needed. Some familiar concrete models are selected to perform Willam’s test in the paper: concrete damaged plasticity and concrete smeared cracking—distributed in the commercial ABAQUS software, scalar damage with coupling to plasticity and isotropic damage—both implemented in the FEAP package. After a brief review of the theory, computations for each model are discussed. Passing or failing Willam’s test by the above models is concluded based on their results, indicating restrictions of their use for finite element computations of concrete structures with predominant mixed-mode fracture.

## 1. Introduction

Sophisticated and complicated simulations for concrete structures are presented in numerous papers. It happens sometimes that it is not convincing whether an employed material model, which can be commonly used, satisfies all basic requirements for proper nonlinear analysis. Appropriate results should actually be guaranteed both for primary stress states and consequently for their complex combinations. First of all, a considered phenomenological model for quasi-brittle materials like concrete should be examined by simple benchmarks illustrating softening/cracking response e.g., for uniaxial tension, also at the point level.

One such benchmark is the so-called Willam’s test which was originally devised in order to study differences between fixed and rotating crack models [1]. This test is still useful for identifying differences between various concrete models in the case when shear induced cracking is important. It has been remarked in [1,2] that the results of particular models can differ even if these models exhibit a similar behavior in uniaxial tension. This tension–shear test with two loading steps is helpful to prevent undesirable effects in more advanced computations because a rotation of principal directions of the strain and stress tensors is very often observed. If a model fails the Willam’s test, then there is no guarantee that it can render properly the structural behavior of concrete elements with predominant mixed-mode fracture. The test itself is quite demanding and even well-known models can fail it, see, e.g., [3].

The main goal of the paper is to study the performance of some popular concrete models in Willam’s test. To the authors’ best knowledge, such a task has not yet been undertaken for the models in question, hence the significance of the research lies in verification of their ability to capture properly the concrete behavior in tension–shear regime.

Concrete damaged plasticity (CDP) and concrete smeared cracking (CSC) models are available in the ABAQUS [4] package. The scalar damage model with the option of weak or strong coupling to hardening plasticity (DAP) as well as the version of isotropic damage (IDA) with the volumetric-deviatoric split are programmed in the FEAP code [5].

**Figure 1:**
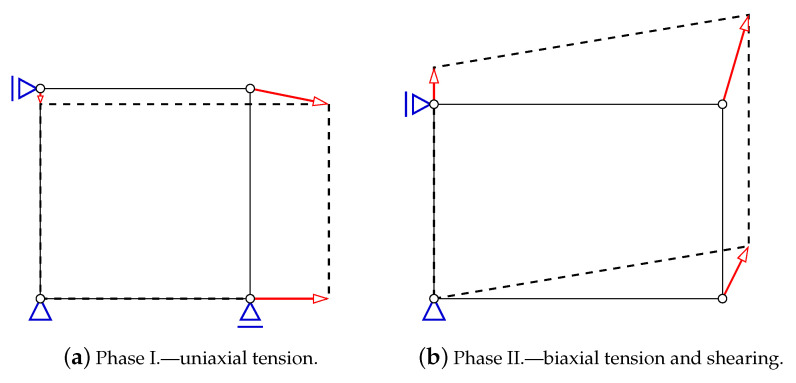
Prescribed displacements for the corresponding strain state in Willam’s test.

The majority of elementary benchmarks with one-phase loading program can be performed and directly confronted with experimental results, e.g., given by [6] for different uniform or scaled, uniaxial or biaxial stress states. The tension–shear test at the point level is computed here, while one finite element (FE) with four nodes is subjected to loading in two steps:

**Phase** **I.**  Uniaxial horizontal tension with vertical contraction due to the Poisson’s effect, according to the relation between the strain increments:
  (1) $$\Delta \epsilon_{11}\;:\;\Delta \epsilon_{22}\;:\;\Delta \gamma_{12}\;=\;1\;:\;-\nu \;:\;0$$
  where $\epsilon_{11}$ and $\epsilon_{22}$ are horizontal and vertical strain components, respectively, $\gamma_{12}$ is shear strain and ν is Poisson’s ratio. In Figure 1a, the scheme of prescribed displacements corresponds to the uniaxial tensile strain state. Such relation is valid until the tensile strength is attained.
**Phase** **II.**  Immediately after the tensile strength is reached, the change of configuration is enforced, Figure 1b. Now, the proportions for the strain increments are arranged in the following way:
  (2) $$\Delta \epsilon_{11}:\Delta \epsilon_{22}:\Delta \gamma_{12}\;=\;0.5:0.75:1$$
  This relation induces tension in two directions with additional shear effect. As a result of such combination, a rotation of principal strain axes occurs; however, tension regime is preserved. At the beginning, the rate of rotation is fast, but, during the evolution, it goes down gradually.

It is suggested by [2] that this two-phased loading process is observed during the analysis of real reinforced concrete (RC) structures. Nevertheless, in the literature, there seem to be no experiments for this or similar tests with rotating principal directions [7]. It turns out that Willam’s test is difficult to be reproduced in a laboratory. However, different authors verified their own proposals in modeling of quasi-brittle materials by means of this numerical test; for example, a comparison of models with respect to multi-surface plasticity is given by [3].

It should be emphasized that Willam’s test is passed if two conditions are satisfied:

**Condition** **1:**  The maximum principal stress is lower than or at most equal to the given uniaxial tensile strength.
**Condition** **2:**  All stress components should converge to zero at the final stage.

Both conditions are physically motivated. Condition 1 is obvious. Concerning Condition 2, the principal strains $\epsilon_{1}$ and $\epsilon_{2}$ have positive values during Phase II. Their directions rotate and in the limit angle $\Theta_{\epsilon }$ between the principal strain $\epsilon_{1}$ and the horizontal strain $\epsilon_{11}$ takes value $52.018^{\circ }$. The test is performed in the plane stress state without confinement, hence material can freely shorten in the out-of-plane direction. Therefore, Willam’s test in Phase II is basically a biaxial tension test with changing directions and no confinement. In such a situation, dilatancy does not appear and in the limit the principal stresses $\sigma_{1}$ and $\sigma_{2}$ should converge to zero, which justifies Condition 2.

In this study, four models (mentioned above) are taken into account. The essential theory for these models is concisely described in Section 2. The paper does not attempt to describe a current development of material models for concrete as well as their more general historical context, so the reader can consult e.g., the following sources on this topic [8,9,10,11,12]. The nonlinear analysis is confined to statics and the assumption of small strains. The main Section 3 shows the corresponding data and the results obtained for each used concrete model. Furthermore, a comparison of diagrams of principal stresses and their directions for these models is shown in Section 4. The last Section 5 contains final conclusions on passing or failing Willam’s test by all the models.

## 2. Overview of Studied Models

### 2.1. Concrete Damaged Plasticity (CDP)

The most popular model to simulate concrete behavior, which is delivered by the ABAQUS software [4], is the concrete damaged plasticity (CDP) model. This model was originally proposed by [13] and then modified by [14].

The theory of non-associated plasticity is linked with isotropic damage, where distinction between stress states for tension and compression is admissible. This nonlinear behavior of the actual body (the material point) is represented by a relationship between stress tensor σ and strain tensor ϵ. Moreover, effective stress tensor $\hat{\sigma }$ is assigned here to the fictitious undamaged counterpart of this body. The above concept is proposed together with the postulate of strain equivalence $\epsilon =\hat{\epsilon }$ [15,16] in the real and effective configurations.

**Figure 2:**
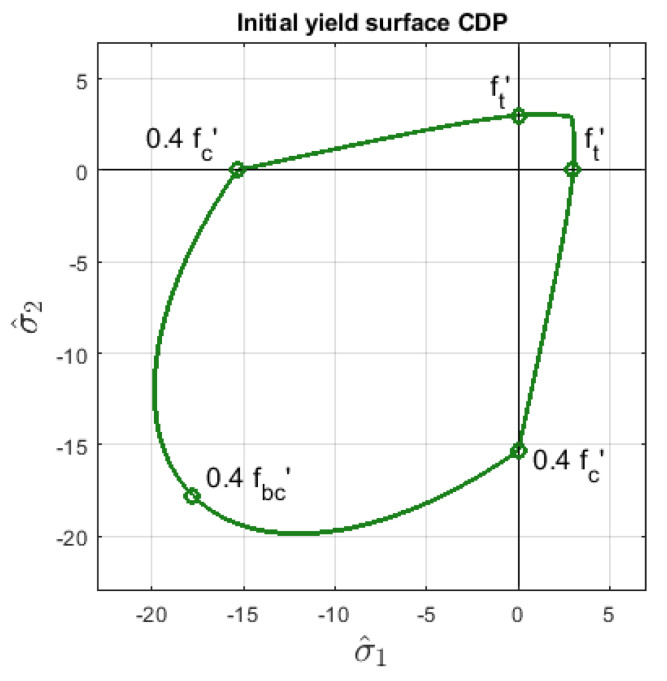
Initial yield surface $F_{\mathrm{CDP}}^{p}$ in 2D effective principal stress space, CDP model, Equation (3), $f_{t}^{\prime }=3$ MPa, $f_{c}=38.3$ MPa, $f_{c}^{\prime }=0.4f_{c}=15.32$ MPa, $f_{bc}^{\prime }=1.16f_{c}^{\prime }=17.77$ MPa.

The yield function is defined in the effective stress space:

(3) $$F_{\mathrm{CDP}}^{p}=\frac{\hat{q}+3\;A\;\hat{p}+B\left({\tilde{\epsilon }}^{\;p}\right)\;\langle {\hat{\sigma }}_{\max}\rangle -C\;\langle -{\hat{\sigma }}_{\max}\rangle }{1-A}-\sigma_{c}\left({\tilde{\epsilon }}_{c}^{\;p}\right)=0$$

where $\hat{q}$ is the effective Mises equivalent stress, $\hat{p}$ is the effective hydrostatic pressure and $\langle {\hat{\sigma }}_{\max}\rangle$ is the positive part of the maximum principal effective stress. Parameters A, B and C decide about the shape of the yield surface; however, ABAQUS users define the following strengths for concrete: the uniaxial tensile strength denoted as $f_{t}^{\prime }$ and $f_{c}$, $f_{c}^{\prime }$, $f_{bc}^{\prime }$ are maximum uniaxial, initial uniaxial, and initial biaxial strengths for compression, respectively. These coefficients affect parameters A, B and C as well as the yield criterion, so that the shape of $F_{\mathrm{CDP}}^{p}$ can be determined. Its initial form in 2D for the data applied in Willam’s test (see Section 3) is shown in Figure 2. This yield surface has first been introduced in [13]. It matches the experimental data well for concrete in stress states with prevailing tension and dominant hydrostatic compression. There are a few other yield surfaces of similar quality widely used for quasi-brittle materials like concrete, cf. [17,18,19].

The current stress state is described via stress–strain relationships separately for uniaxial compression and uniaxial tension:

(4) $$\sigma_{c}=\sigma_{c}\left({\tilde{\epsilon }}_{c}^{\;p}\right)\;\mathrm{and}\;\sigma_{t}=\sigma_{t}\left({\tilde{\epsilon }}_{t}^{\;p}\right)$$

The primary argument of the above yield function is equivalent plastic strain ${\tilde{\epsilon }}^{\;p}$, split into ${\tilde{\epsilon }}_{c}^{\;p}$ for compression and ${\tilde{\epsilon }}_{t}^{\;p}$ for tension. Of course, the standard additive decomposition of strain rate into elastic and plastic parts is assumed in the CDP model:

(5) $$\dot{\epsilon }={\dot{\epsilon }}^{e}+{\dot{\epsilon }}^{p}$$

**Figure 3:**
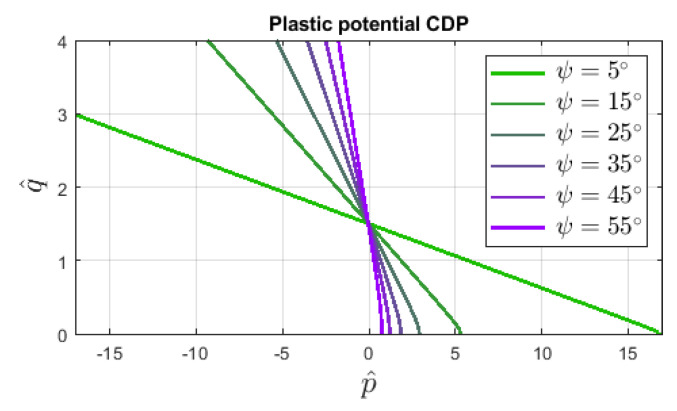
Influence of dilatancy angle ψ in the shape of plastic potential surfaces $G_{\mathrm{CDP}}^{p}$ defined in $\hat{p}-\hat{q}$ plane, CDP model, Equation (6), $f_{t}^{\prime }=3$ MPa, E=0.1.

More in-depth description concerning the determination of nonlinear functions for compressive crushing and tensile cracking can be found e.g., in [4,20,21]. The plastic flow potential function is determined in the following way:

(6) $$G_{\mathrm{CDP}}^{p}=\sqrt{{\left(\tan\psi \;E\;f_{t}^{\prime }\right)}^{2}+{\hat{q}}^{2}}\;+\;\tan\psi \;\hat{p}$$

where ψ is the dilatancy angle and the so-called eccentricity E decides about the shape of the tip of surface $G_{\mathrm{CDP}}^{p}$. Figure 3 illustrates the influence of dilatancy angle ψ on function $G_{\mathrm{CDP}}^{p}$ described in the $\hat{p}$-$\hat{q}$ plane. Different aspects of the dilatancy definition connected with the plastic potential are addressed e.g., by [22]. It should be noticed that both functions $F_{\mathrm{CDP}}^{p}$ and $G_{\mathrm{CDP}}^{p}$ are formulated as modifications of the classical Burzyński–Drucker–Prager (BDP) yield criterion.

If damage phenomenon is additionally activated in this plasticity-based model to consider a stiffness degradation of concrete, then the stress σ for the real material structure corresponds to the effective stress $\hat{\sigma }$ in the undamaged skeleton of the body via the damage parameter ω. It should also be recalled that ω evolves from 0 for the intact material to 1 for a full loss of stiffness. The constitutive relationship can be written in the form:

(7) $$\sigma =\left(1-\omega \right)\;\hat{\sigma }=\left(1-\omega \right)\;D:\left(\epsilon -\epsilon^{p}\right)$$

where D is the fourth order tensor for the initial elastic stiffness. In a similar fashion to the distinction of uniaxial stress–strain relations in compressive and tensile regimes, the stiffness degradation as well as its recovery factors can be determined independently. Damage ω is split in such way:

(8) $$1-\omega =\left[1-s_{c}\left(\hat{\sigma }\right)\;\omega_{t}\right]\;\left[1-s_{t}\left(\hat{\sigma }\right)\;\omega_{c}\right]$$

Variable $\omega_{t}$ reduces the stiffness in tension and consequently $\omega_{c}$—in compression, while functions $s_{c}\left(\hat{\sigma }\right)$ and $s_{t}\left(\hat{\sigma }\right)$ are responsible for stiffness recovery. A wider discussion of the damage definition in the CDP model and of the crack closing phenomenon can be found in [23].

This model in the ABAQUS/Standard version is equipped with the regularization by the viscous term for the scalar plastic strain rate and also for the rate of degradation, according to the generalized approach of [24]. The sensitivity analysis of the viscosity parameter is performed e.g., by [25]. However, the regularization is not regarded at the material point level, so the CDP model has no viscosity in Willam’s test and relaxation time is equal to zero.

### 2.2. Concrete Smeared Cracking (CSC)

Using the ABAQUS/Standard version for simulations of concrete structures another nonlinear model can also be employed. It is called the concrete smeared cracking (CSC) model [4] and allows one to compute the problem under monotonic loading. Hence, it works in the nonlinear analysis of quasi-brittle materials but without the crack closing effect.

The concept of smeared cracking in concrete comes from the late sixties, cf. [26]. In the CSC model, crack orientation is normal to maximum principal stress $\sigma_{I}$ reaching tensile strength $f_{t}^{\prime }$ and then held on fixed. Consequently, the elastic stiffness becomes reduced. In the region of predominant tensile stress, a yield surface $F_{\mathrm{CSC},t}^{p}$ is introduced, called here the crack detection surface:

(9) $$F_{\mathrm{CSC},t}^{p}=\bar{q}+3\;\bar{p}+T\frac{{\tilde{\sigma }}_{t}}{f_{t}^{\prime }}\left(\frac{{\tilde{\sigma }}_{t}}{3}-\bar{p}\right)-2\;{\tilde{\sigma }}_{t}=0$$

where $\bar{q}$ and $\bar{p}$ are introduced similar to $\hat{q}$ and $\hat{p}$, but without stress components of σ associated with open cracks—this way, secondary cracks can form only perpendicular to already existing ones (no more than two cracks perpendicular to each other in 2D case). Relation ${\tilde{\sigma }}_{t}\left(\lambda_{t}\right)$ for uniaxial tension governs softening of the yield surface and parameter T influences the shape of the yield surface. In order to control softening of function $F_{\mathrm{CSC},t}^{p}$, the classical formalism of the associated elastoplasticity is introduced with strain rate decomposition:

(10) $$\dot{\epsilon }={\dot{\epsilon }}^{e}+{\dot{\epsilon }}_{t}^{p}$$

and the associated flow rule:

(11) $${\dot{\epsilon }}_{t}^{p}={\dot{\lambda }}_{t}\;\frac{\partial F_{t}^{p}}{\partial \sigma }$$

In the above equation, $\epsilon_{t}^{p}$ is the plastic strain tensor for the crack detection and $\lambda_{t}$ is the tensile plastic multiplier.

It has to be stressed that the elastoplastic formulation described above is only used for a proper description of softening of the yield surface $F_{\mathrm{CSC},t}^{p}$. Immediately after crack detection, a damage elasticity approach is introduced linking total values of stress and strain tensors according to the formula:

(12) $$\sigma =D^{\mathrm{cr}}:\epsilon$$

where $D^{\mathrm{cr}}$ is a secant elastic operator formulated in the spatially fixed coordinate frame *n*,*t*,*s* aligned with the crack plane (with axis *n* normal to this plane). The normal component of the stiffness operator $D^{\mathrm{cr}}$ in direction *n* is defined as:

(13) $$D_{nnnn}^{\mathrm{cr}}=\frac{\sigma_{nn}}{\epsilon_{nn}}$$

where the value of the stress $\sigma_{nn}$ for a given value of total strain $\epsilon_{nn}$ is computed using the stress–strain relation for uniaxial tension, a so-called *tension stiffening curve* [4], defined by a user. Additionally, Poisson’s effect is neglected for an active crack leading to the formula:

(14) $$D_{nntt}^{\mathrm{cr}}=D_{nnss}^{\mathrm{cr}}=0$$

The shear stiffness decreases for cracking, so Kirchhoff (shear) modulus *G* is reduced according to the shear retention concept [1,27]:

(15) $$D_{ntnt}^{\mathrm{cr}}=D_{nsns}^{\mathrm{cr}}=\rho \;G$$

**Figure 4:**
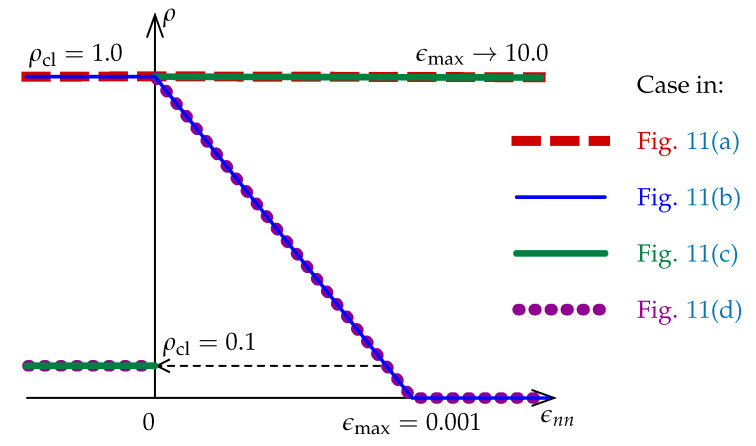
Functions ρ for shear retention effect used in the CSC model (Section 3.2).

Function $\rho (\epsilon_{nn})$ is illustrated in Figure 4 for cases computed in Section 3.2 and defined as follows:

(16) $$\rho =\left\{\begin{array}{cc} \rho_{\mathrm{cl}} & \mathrm{for}\;\epsilon_{nn}<0\\ 1-\frac{\epsilon_{nn}}{\epsilon_{\max}}\; & \mathrm{for}\;0\leq \epsilon_{nn}<\epsilon_{\max}\\ 0 & \mathrm{for}\;\epsilon_{nn}\geq \epsilon_{\max} \end{array}\right.$$

In the region of predominant compressive stress in the CSC model, the standard associated elastoplastic approach is used with the compressive yield surface in the classical BDP form:

(17) $$F_{\mathrm{CSC},t}^{p}=\hat{q}+\sqrt{3}\left(S\;\hat{p}-\tau_{c}\right)=0$$

where S is a material parameter specified by the ratio of biaxial to uniaxial compressive strengths and hardening/softening of the yield surface is governed by the cohesion function $\tau_{c}\left(\lambda_{c}\right)$. The standard strain rate elastoplastic additive decomposition is adopted:

(18) $$\dot{\epsilon }={\dot{\epsilon }}^{e}+{\dot{\epsilon }}_{c}^{p}$$

but now the associated plastic flow is:

(19) $${\dot{\epsilon }}_{c}^{p}={\dot{\lambda }}_{c}\left[1+W{\left(\frac{\hat{p}}{\sigma_{c}}\right)}^{2}\right]\frac{\partial F_{c}^{p}}{\partial \sigma }$$

where $\epsilon_{c}^{p}$ is the plastic strain connected with compression, $\lambda_{c}$ is the compressive plastic multiplier, parameter W depends on the ratio of stress components for biaxial and uniaxial compression as well as the ratio of respective strains, and $\sigma_{c}\left(\epsilon_{c}\right)$ is the hardening/softening curve for uniaxial compression, cf. Equation (4)${}_{1}$.

**Figure 5:**
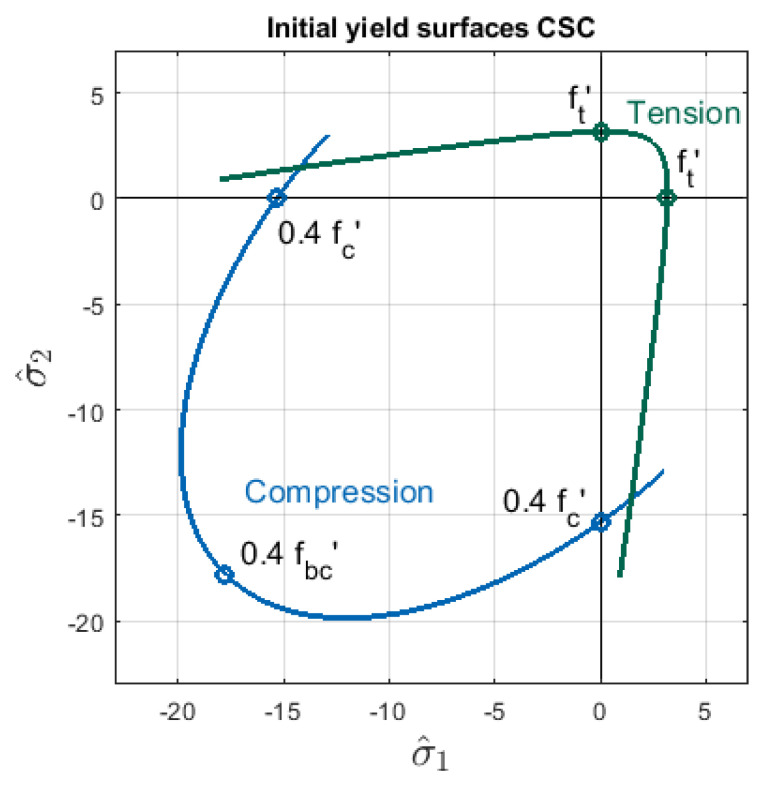
Initial yield surfaces: $F_{\mathrm{CSC},t}^{p}$ for tension given in Equation (9) and $F_{\mathrm{CSC},c}^{p}$ for compression given in Equation (17), defined in 2D effective principal stress space, CSC model, introduced values of strengths as for $F_{\mathrm{CDP}}^{p}$ in Figure 2.

The diagram $\sigma_{c}\left(\epsilon_{c}\right)$ serves as well to define the cohesion function $\tau_{c}\left(\lambda_{c}\right)$—the details are given in [4]. The compressive yield surface $F_{\mathrm{CSC},t}^{p}$ as well the crack detection surface $F_{\mathrm{CSC},t}^{p}$ in their initial state are presented in Figure 5 based on the data for Willam’s test (see Section 3). Hence, the yield criterion is specified in the stress space and consists of two different surfaces similarly to [28].

As can be seen from the above description that the CSC model belongs to the family of total smeared fixed crack models [1,27,29], enhanced by the standard elastoplastic formulation for compressive stress. More detailed explanations for the CSC model, e.g., how to recalculate values of parameters T, W and S from the data entered by users are included in [4]. It should be mentioned that, for this model, mesh sensitive results can occur. Therefore, if the CSC model is applied in the computations of concrete structures, then the crack band theory [30] should be employed to set a proper size of the finite element corresponding to the expected cracking zone, see also [31,32]. Wide discussion concerning smeared cracking models can be found e.g., in [27,29]. Moreover, the concept of rotating crack model has also been included in the Modified Compression Field Theory (MCFT) developed for a combined description of cracked concrete and reinforcement at the material point level, see [33]. However, in that case, the descending branch of the stress–strain curve for concrete represents the tension stiffening phenomenon for reinforced concrete (RC) rather than the tension softening for plain concrete.

### 2.3. Damage-Plasticity (DAP)

A combination of the damage theory defined in strain space with hardening plasticity given in stress space leads to the damage-plasticity (DAP) model. The model presented here is implemented in the FEAP package [5] and is based on works of [16,34,35].

Starting from the pure scalar damage model, one damage measure ω is introduced in the classical way. The effective stress $\hat{\sigma }$ is distinguished as in the CDP model:

(20) $$\sigma =\left(1-\omega \right)\;\hat{\sigma }=\left(1-\omega \right)\;D:\epsilon^{e}$$

**Figure 6:**
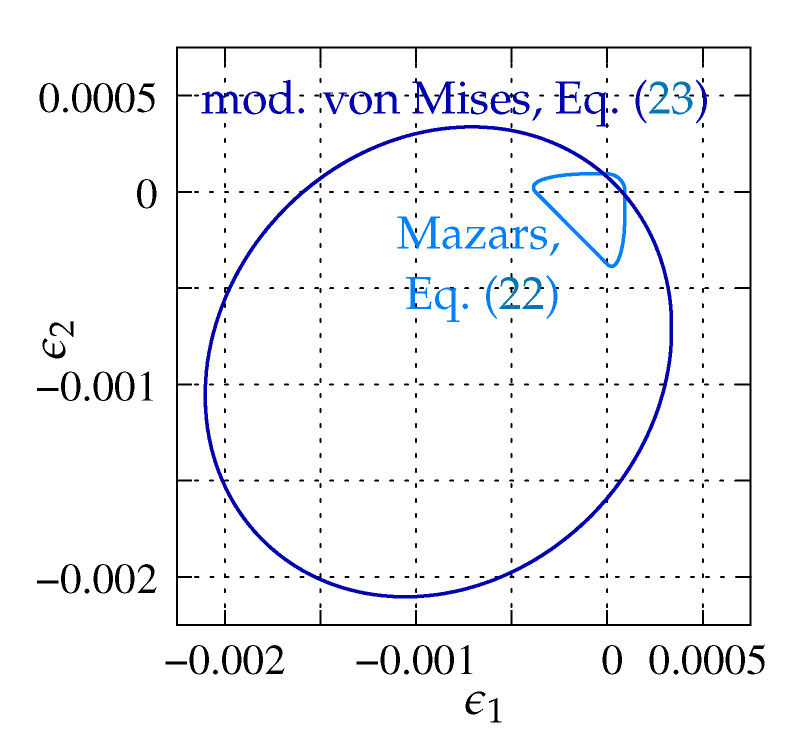
Illustration of equivalent strain measure definitions in 2D principal strain space, plane stress conditions, ν=0.2, $\tilde{\epsilon }=0.00009375$, k=12.7667.

The assumption of strain equivalence also remains valid. Damage is characterized by the following loading function:

(21) $$F_{\mathrm{DAP}}^{d}=\tilde{\epsilon }-\kappa^{d}=0$$

where $\tilde{\epsilon }\left(\epsilon \right)$ is an equivalent strain measure and $\kappa^{d}$ is a damage history parameter. This damage activation function $F_{\mathrm{DAP}}^{d}$ controls the behavior of the material after the damage threshold $\kappa_{o}$ is attained during the loading history. The equivalent strain measure $\tilde{\epsilon }$, which should demonstrate different behavior in tension and compression, can be defined for instance according to the idea by [36]:

(22) $$\tilde{\epsilon }=\sqrt{\sum_{I=1}^{3}{\langle \epsilon_{I}\rangle }^{2}}$$

where $\langle \epsilon_{I}\rangle$ is the positive part of *I*-th principal strain $\epsilon_{I}$. The second proposal, employed in the DAP model, is the modified von Mises definition [37]:

(23) $$\tilde{\epsilon }=\frac{\left(k-1\right)\;I_{1}^{\epsilon }}{2k\;(1-2\nu )}+\frac{1}{2k}\sqrt{{\left(\frac{k-1}{1-2\nu }I_{1}^{\epsilon }\right)}^{2}+\frac{12\;k\;J_{2}^{\epsilon }}{{\left(1+\nu \right)}^{2}}}$$

where $I_{1}^{\epsilon }$ is the first strain tensor invariant, $J_{2}^{\epsilon }$ is the second deviatoric strain invariant, ν is the Poisson’s ratio and $k=f_{c}/f_{t}^{\prime }$ is the ratio between compressive and tensile strength. Both functions are depicted in Figure 6 according to the data used in Willam’s test (see Section 3). The damage growth function directly depends on the damage history parameter $\kappa^{d}$ and can be determined e.g., as the exponential softening relation. It properly reproduces tensile fracture phenomenon in concrete by the asymptotic function [38]:

(24) $$\omega \left(\kappa^{d}\right)=1-\frac{\kappa_{o}}{\kappa^{d}}\left(1-\alpha +\alpha e^{-\eta (\kappa^{d}-\kappa_{o})}\right)$$

Parameter α corresponds to the residual stress $\left(1-\alpha \right)\;E\;\kappa_{o}$, so, if α=1, the total loss of the stiffness is attained. The ductility parameter η is connected with the rate of softening and the concrete fracture energy $G_{f}$. The elastic constant *E* is Young’s modulus.

During unloading, the secant stiffness (1−ω)D results in a return to the origin, i.e., no residual strains are observed and damage does not grow. The damage-based model can be coupled to plasticity in order to include physically observed irreversible strains.

The yield function for the plastic component in the DAP model is formulated in the effective stress space:

(25) $$F_{\mathrm{DAP}}^{p}=\tilde{\sigma }-\sigma_{y}=0$$

where $\tilde{\sigma }\left(\hat{\sigma }\right)$ is an equivalent measure of effective stress and $\sigma_{y}\left(\kappa^{p}\right)$ is an isotropic hardening law with the yield strength limit. Function $F_{\mathrm{DAP}}^{p}$ can be described, for example, by the Huber–Mises–Hencky (HMH) or BDP criteria. Proportionality between the plastic multiplier $\dot{\lambda }$ and the plastic strain measure $\kappa^{p}$ is assumed. The plastic multiplier λ determines the magnitude of plastic strains $\epsilon^{p}$ according to the non-associated flow rule:

(26) $${\dot{\epsilon }}^{p}=\dot{\lambda }\;m=\dot{\lambda }\;\frac{\partial G_{\mathrm{DAP}}^{p}}{\partial \hat{\sigma }}$$

where *m* is the plastic flow direction and $G_{\mathrm{DAP}}^{p}$ is a plastic potential function. Based on the standard additive decomposition (5) and considering the plastic consistency condition, the elastic strain rate is written as:

(27) $${\dot{\epsilon }}^{e}=\dot{\epsilon }-\frac{1}{h}m⊗n:\dot{\hat{\sigma }}$$

where *h* is the hardening (or softening) modulus and the gradient tensor $n=\partial F_{\mathrm{DAP}}^{p}/\partial \hat{\sigma }$ is associated with the yield function $F_{\mathrm{DAP}}^{p}$. Finally, using the Sherman–Morrison formula, the tangential relation is derived:

(28) $$\dot{\hat{\sigma }}=D^{\mathrm{ep}}:\dot{\epsilon }$$

with the elastoplastic tensor:

(29) $$D^{\mathrm{ep}}=D-\frac{D:m⊗n:D}{h+n:D:m}$$

It is seen that, in the DAP model, the coupling of damage and plasticity is given in Equation (20). The stress rate during the evolution of damage and plasticity can be computed as:

(30) $$\dot{\sigma }=\left(1-\omega \right)\;\dot{\hat{\sigma }}-\dot{\omega }\;\hat{\sigma }$$

The rate of damage during loading ($\kappa^{d}=\tilde{\epsilon }$) is calculated in the way:

(31) $$\dot{\omega }=\frac{d\omega }{d\kappa^{d}}\;\frac{d\kappa^{d}}{d\tilde{\epsilon }}\;\frac{\partial \tilde{\epsilon }}{\partial \epsilon }:\dot{\epsilon }$$

and during unloading $\dot{\omega }=0$. The stress–strain relation for the coupled model is:

(32) $$\dot{\sigma }=\left[\left(1-\omega \right)\;D^{\mathrm{ep}}-L\;\hat{\sigma }⊗s\right]:\dot{\epsilon }$$

where the following definitions are used:

(33) $$L=\frac{d\omega }{d\kappa^{d}}\;\;\;s=\frac{\partial \tilde{\epsilon }}{\partial \epsilon }$$

There are two possibilities of coupling in the DAP model. The equivalent strain $\tilde{\epsilon }$ can depend on the total strain tensor ϵ or its elastic part $\epsilon^{e}$. If the full coupling via $\tilde{\epsilon }(\epsilon )$ is employed, then the plastic strains also stimulate the damage growth. When the second option $\tilde{\epsilon }(\epsilon^{e})$ is selected, the coupling effect is weaker, and it seems to be more relevant in the modeling of quasi-brittle materials.

Beyond the point level, the DAP model can be enhanced to a nonlocal version by means of a gradient-type or an integral-type approach in order to ensure mesh-objective results, see, e.g., [35,39,40,41,42].

### 2.4. Isotropic Damage (IDA)

Material (elastic stiffness) degradation can be introduced into the model using a fourth-order damage tensor or a second-order damage tensor for anisotropic description, see, e.g., [43,44]. The decomposition of damage effect into different tensile and compressive actions is described by the CDP model presented above or for instance by [38,45,46,47]. Remaining within the isotropic description a volumetric-deviatoric split with two damage variables and one equivalent strain measure can also be applied by [48].

Even a simpler upgrade of the scalar damage theory is originated in [49], where one damage parameter influences a different decrease of stiffness for bulk modulus *K* and shear (Kirchhoff) modulus *G*. Moreover, the magnitudes of degradation of *K* and *G* depend on the sign of dilatancy. This approach will be called the isotropic damage (IDA) model here. Firstly, the elasticity operator D is written as:

(34) D=KI⊗I+2GQ

where *I* is the second order identity tensor, Q=I−I⊗I/3 is a fourth order tensor and I is the fourth order identity tensor. Combining Equations (20) and (34), the following constitutive equation is obtained:

(35) σ=(1−ω)KI⊗I:ϵ+(1−ω)2GQ:ϵ

Now, the deviatoric strain is $\epsilon_{\mathrm{dev}}=Q:\epsilon$ and the dilatancy is θ=I:ϵ, so Equation (35) is rewritten:

(36) $$\sigma =\left(1-\omega \right)\;K\;I\;\theta +\left(1-\omega \right)\;2G\;\epsilon_{\mathrm{dev}}$$

Referring to [49], where the anisotropic damage description is shown, positive and negative parts of hydrostatic pressure and stress components are distinguished. It is indicated that damage is connected with a micro-defect (micro-crack) pattern which evolves in a different way under tension and under compression. The action of damage can be reduced for the negative (compressive) effective stress and the volumetric part of stiffness when the material is subjected to compression. The influence of damage in the IDA model is governed by dilatancy θ, so two cases are considered.

If θ>0, then Equation (36) is expressed as:

(37) $$\sigma ={\left(1-\omega \right)}^{P}\;K\;I\;\theta +\left(1-\omega \right)\;2G\;\epsilon_{\mathrm{dev}}$$

where the power P regulates damage of a volumetric-deviatoric split. In fact, P>1.0 accelerates the damage progress for the bulk modulus. If P=1.0, then pure scalar damage is retrieved. It is possible P<1.0, but, in that case, the development of volumetric degradation is slowed down. The rate of stress is:

(38) $$\dot{\sigma }={\left(1-\omega \right)}^{P}\;K\;I\;\dot{\theta }+\left(1-\omega \right)\;2G\;{\dot{\epsilon }}_{\mathrm{dev}}-\left[P\;{\left(1-\omega \right)}^{P-1}\;K\;I\;\theta +2G\;\epsilon_{\mathrm{dev}}\right]\;\dot{\omega }$$

If θ<0, Equation (36) is redefined:

(39) $$\sigma ={\left(1-R\;\omega \right)}^{P}\;K\;I\;\theta +\left(1-\omega \right)\;2G\;\epsilon_{\mathrm{dev}}$$

where R∈[0.0,1.0] is a damage reduction factor. The closer to 0 the factor is, the less degradation of the bulk stiffness is involved. It should be noted that, in [50], a similar relation is introduced with P=1.0 and R=0.0, i.e., the bulk stiffness remains elastic for the negative dilatancy. Accordingly, for linearization, the rate of stress should be written as:

(40) $$\dot{\sigma }={\left(1-R\;\omega \right)}^{P}\;K\;I\;\dot{\theta }+\left(1-\omega \right)\;2G\;{\dot{\epsilon }}_{\mathrm{dev}}-\left[P\;R\;{\left(1-R\;\omega \right)}^{P-1}\;K\;I\;\theta +2G\;\epsilon_{\mathrm{dev}}\right]\;\dot{\omega }$$

The IDA model can also be implemented as nonlocal to prevent spuriously sensitive mesh discretization, cf. [49,51].

## 3. Testing of Considered Models

The overall data for each concrete model are as in [3]. The elastic constants are as follows: Young’s modulus E=32000 MPa and Poisson’s ratio ν=0.20. The next parameters of the particular model are tuned to initial uniaxial tensile strength $f_{t}^{\prime }=3$ MPa, maximum uniaxial compressive strength $f_{c}=38.3$ MPa, and tensile fracture energy $G_{f}=0.11$ N/mm. Small strains and plane stress conditions are assumed. The finite element size is equal to 100 mm. As mentioned in the Introduction, it is expected that the response of each model can vary despite the same set of global data. Furthermore, crucial features of the models are exposed in the presentation of results.

### 3.1. Concrete Damaged Plasticity (CDP)

**Figure 7:**
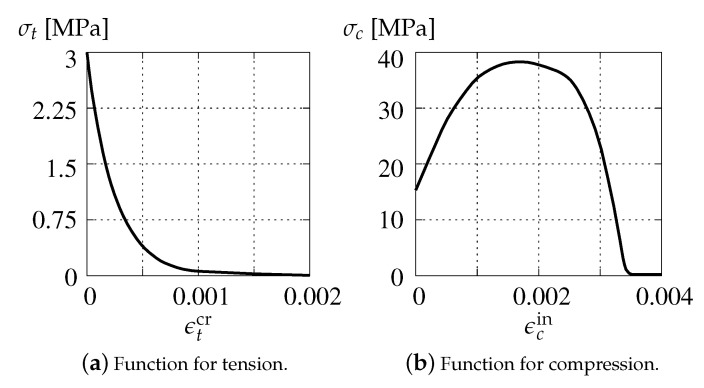
Material stress–strain relations for CDP model.

In the CDP model, stress–strain relations have to be defined separately for tensile cracking and compressive crushing. For softening in the tension regime, starting from the point where a so-called cracking strain $\epsilon_{t}^{\mathrm{cr}}=0.0$ (i.e., difference of total and elastic strains) corresponds to the tensile strength $f_{t}^{\prime }=3.0$ MPa, the curvilinear relationship between the uniaxial tensile stress $\sigma_{t}$ and the cracking strain $\epsilon_{t}^{\mathrm{cr}}$ is determined as shown in Figure 7a. When uniaxial compression is considered, the stress-inelastic strain function similar to a parabola, cf. [52,53], is employed. Maximum compressive strength $f_{c}=38.3$ MPa is adopted for inelastic strain $\epsilon_{c}^{\mathrm{in}}=0.00172$. This diagram is depicted in Figure 7b.

The initial compressive strength is $f_{c}^{\prime }=15.32$ MPa, which fits $0.4\;f_{c}$. The ratio of the biaxial compressive stress to the uniaxial compressive stress is 1.16, so that the initial biaxial compressive strength $f_{bc}^{\prime }$ is equal to 17.77 MPa. The initial yield surface $F_{\mathrm{CDP}}^{p}$ is drawn in Figure 2 in 2D effective principal stress space. The ratio of equivalent stress $\hat{q}$ on the tensile and compressive meridians is $K_{c}$ and the default value $\frac{2}{3}$ is used in the computations, see also [4].

For the CDP model without activated damage, the influence of dilatancy angle ψ is studied; hence, it can range from $5^{\circ }$ to $55^{\circ }$. The plastic potential functions $G_{\mathrm{CDP}}^{p}$ for different angles are compared in Figure 3 with the same eccentricity E=0.1. This default value is assumed in the numerical analysis.

**Figure 8:**
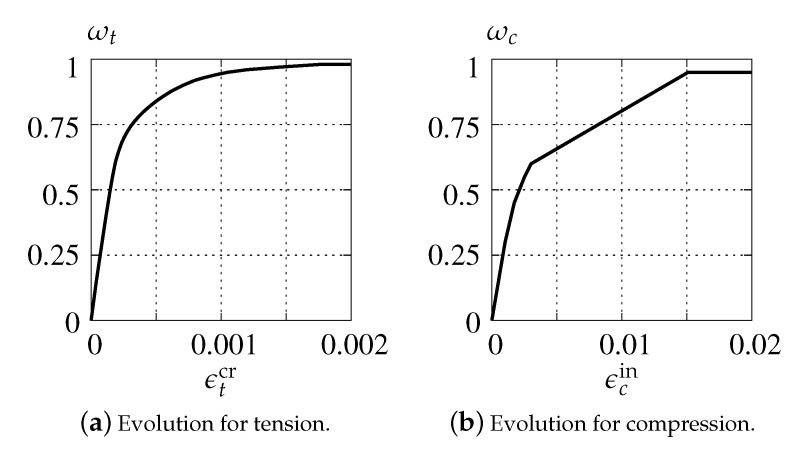
Damage growth functions for CDP model.

In the case of activation of damage growth functions for tension and compression as shown in Figure 8, the test is run only for dilatancy $\psi =25^{\circ }$. Moreover, damage $\omega_{c}$ is insignificant in Willam’s test, where compression is not present.

**Figure 9:**
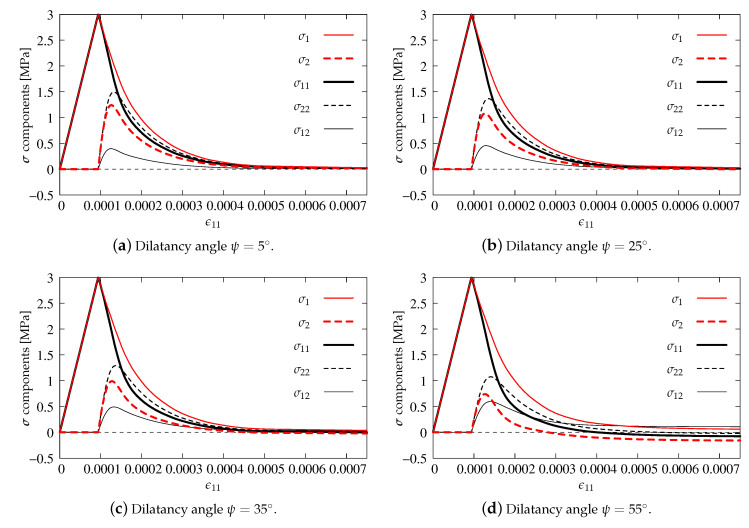
Comparison of stress components for CDP model—pure plasticity.

**Figure 10:**
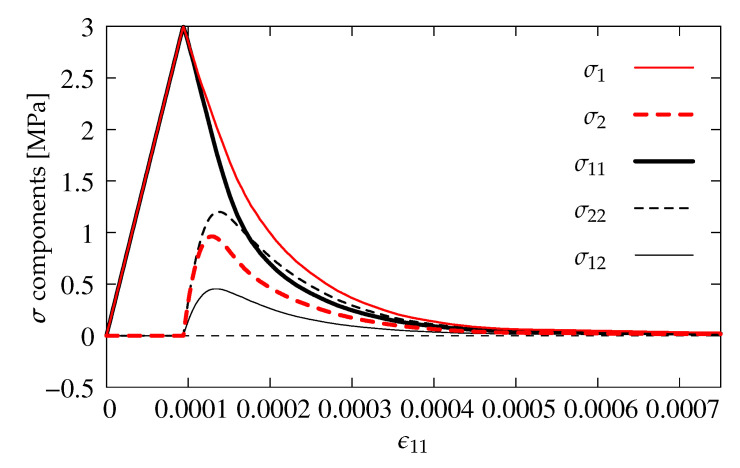
Comparison of stress components for CDP model—plasticity with damage, dilatancy angle $\psi =25^{\circ }$.

Figure 9 and Figure 10 show stress components $\sigma_{11}$, $\sigma_{22}$ and $\sigma_{12}$ together with their principal values $\sigma_{1}$, $\sigma_{2}$ versus axial strain $\epsilon_{11}$.

First of all, the influence of different dilatancy angles ψ in the plasticity model is verified. In the computations, the same values of ψ=5, 25, 35 and $55^{\circ }$ as for the simulation of punching shear presented by [22] are introduced. It is clearly visible that, for each option, the values of all stress components are at most equal to the uniaxial tensile strength $f_{t}^{\prime }=3$ MPa, so the first condition of Willam’s test is satisfied. It is also noticed that the stresses decrease in the second phase of the test. For $\psi =5^{\circ }$ and $\psi =25^{\circ }$ (see Figure 9a,b and Figure 10), they approach zero at the final stage; hence, the second condition of Willam’s test is also fulfilled. However, when the dilatancy angle ψ in the CDP model becomes larger than $35^{\circ }$, negative values of horizontal stress $\sigma_{11}$ and minimum principal stress $\sigma_{2}$ are observed. It is evidently demonstrated in Figure 9d for $\psi =55^{\circ }$. Therefore, for the CDP model, a similar observation as in [22] can be made, i.e., the dilatancy angle ψ should not be larger than about $35^{\circ }$. Larger values produce a dilatancy effect (manifested by negative stresses), which could be justifiable in confinement conditions as e.g., in [54], but for Willam’s test is simply non-physical and violates Condition 2.

Additionally, the results illustrated in Figure 10 are prepared for the case where also damage functions are incorporated in the CDP model. The diagrams are similar to the one presented in Figure 9b. The difference is noticed only for $\sigma_{22}$ and $\sigma_{2}$, where activated damage reduces their maximum values. Indeed, the presence of damage in this model for Willam’s test is hardly relevant. It should be recalled that damage in the CDP model is basically introduced to simulate a stiffness degradation during unloading. Moreover, it occurs (results not included in the paper) that only damage evolution for tension, cf. Figure 8, is able to modify the solution.

### 3.2. Concrete Smeared Cracking (CSC)

The material relations in the CSC model are determined identically as in the CDP model. Figure 7a shows the softening branch of tensile stress $\sigma_{t}$ as the function of cracking strain $\epsilon_{t}^{\mathrm{cr}}$, while Figure 7b depicts the relationship of compressive stress $\sigma_{t}$ and inelastic strain $\epsilon_{c}^{\mathrm{in}}$. The associated strengths are also the same, but now two initial yield surfaces $F_{\mathrm{CSC},t}^{p}$ for tension and $F_{\mathrm{CSC},t}^{p}$ for compression are combined to obtain the yield criterion, see Figure 5.

ABAQUS users can decide about the shape of both surfaces as well as the values of parameters T, W, and S related to them via so-called failure ratios [4]:

- the ratio of the biaxial compressive stress to the uniaxial compressive stress equals 1.16 as the default value,
- the ratio of the uniaxial tensile strength $f_{t}^{\prime }$ to the maximum uniaxial compressive strength $f_{c}$ equals 0.078329 for Willam’s test,
- the ratio of the principal plastic strain $\epsilon_{1}^{p}$ for biaxial and uniaxial compression, respectively, equals 1.28 as the default value,
- the ratio of the tensile principal stress $\sigma_{1}$ at cracking, when $\sigma_{2}$ is at the ultimate compressive stress, to the tensile cracking stress for uniaxial tension equals $\frac{1}{3}$ as the default value.

It should be emphasized that the associated flow is assumed in the CSC model. If no more options are stated, then full shear retention is given as the default. In the case of detailed specification of the shear retention option, the following parameters are defined: fraction $\rho_{\mathrm{cl}}$ of shear modulus *G* for closed cracks in concrete (the default value is 1.0) and the maximum value of the strain $\epsilon_{nn}$ normal to the crack plane is $\epsilon_{\max}$—cf. Figure 4 (the default value is a large number).

**Figure 11:**
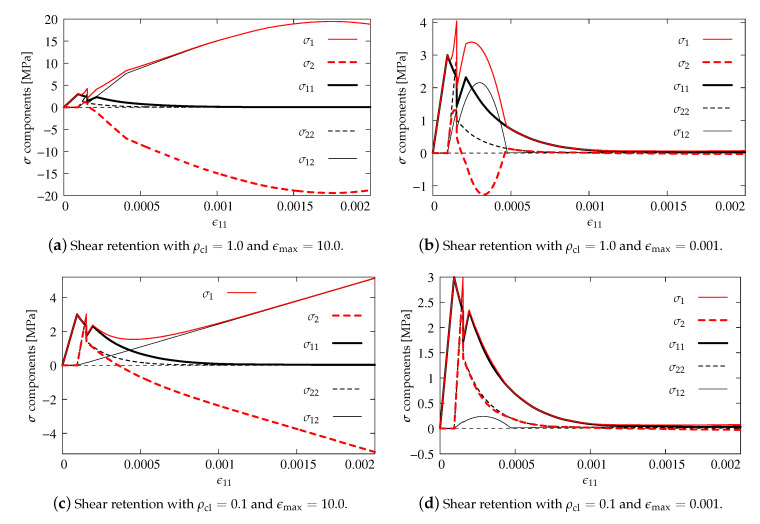
Comparison of stress components for the CSC model.

**Figure 12:**
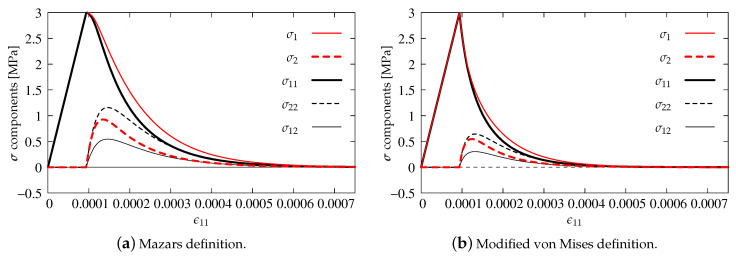
Comparison of stress components for DAP model—pure damage.

The shear retention effect is analyzed in the computations for the CSC model. Analogically to the previous subsection, all stress components are depicted depending on strain $\epsilon_{11}$. Figure 11a shows results for $\rho_{\mathrm{cl}}=1.0$ and $\epsilon_{\max}=10.0$, which corresponds to full shear retention. This value of $\epsilon_{\max}$ for $\epsilon_{11}=0.002$ gives ρ=0.9998≈1.0. It is visible that not only tensile strength $f_{t}^{\prime }=3$ MPa is exceeded many times by the maximum principal stress $\sigma_{1}$ and shear stress $\sigma_{12}$, but they do not converge to zero stress. The minimum principal stress $\sigma_{2}$ from about $\epsilon_{11}=0.0002$ becomes negative and further makes a mirror image of $\sigma_{1}$ relative to the horizontal axis for zero stress. For the case with $\rho_{\mathrm{cl}}=1.0$ and much smaller $\epsilon_{\max}=0.001$, the effects of shear retention are partly active, see Figure 11b. Of course, formation of the first crack is correlated with the first peak when $\sigma_{1}=f_{t}^{\prime }=3$ MPa, but, after that, the maximum value of $\sigma_{1}$ is larger than 4 MPa at the moment of formation of the second crack. Next, a sudden drop is noticeable in the diagrams of stress components. Both the increase of values of $\sigma_{1}$ and $\sigma_{12}$ as well as the decrease of $\sigma_{2}$ (negative values occur again) are connected with shear retention. For $\epsilon_{11}\approx 0.0005$, this effect disappears, but the principal stresses rather deviate than go to zero stress. The results for the opposite case with small $\rho_{\mathrm{cl}}=0.1$ and large $\epsilon_{\max}=10.0$ are presented in Figure 11c. Again, the formation of two cracks is observed, but further shear retention makes that $\sigma_{1}$ and $\sigma_{12}$ go to plus infinity, while $\sigma_{2}$ to minus infinity. The diagram of minimum principal stress $\sigma_{2}$ looks like a mirror image (with the negative sign) of the diagram of maximum principal stress $\sigma_{1}$. Figure 11d illustrates the diagrams of stress components for the option with small $\rho_{\mathrm{cl}}=0.1$ and also small $\epsilon_{\max}=0.001$. As previously, the crack formation is noticed and moreover the value of $f_{t}^{\prime }=3$ MPa is not exceeded, the presence of shear retention is almost imperceptible, but finally principal stresses $\sigma_{1}$ and $\sigma_{2}$ depart from zero value. Figure 11a and Figure 12a indicate that the parameter $\rho_{\mathrm{cl}}$ plays an important role in the tension regime, and its small value can lead to a substantial reduction of stress $\sigma_{12}$. This observation is at odds with the CSC model description given in [4], according to which the parameter $\rho_{\mathrm{cl}}$ should have no influence on the behavior of an open crack as it defines only the shear retention factor for a closed crack (in compression). It can be concluded that the actual implementation of the CSC model in the ABAQUS package departs at this point from its description [4]. The shear retention effect is suppressed for small $\rho_{\mathrm{cl}}=0.1$. Summarizing, the CSC model fails Willam’s test, although the results for the last considered case pass the first condition of the test. In fact, the behavior of the CSC model is very similar to the behavior of the standard fixed smeared crack model as observed by [1,27,29]. For both these models, the source of non-physical behavior seen in Figure 11a and Figure 12a is quite obvious and has already been identified in [1]—retaining the full stiffness for shear in Phase II leads to the unbounded increase of shear stress for increasing shear strain.

### 3.3. Damage-Plasticity (DAP)

In damage theory, the threshold is calculated as quotient of the uniaxial tensile strength and Young’s modulus, so $\kappa_{o}=f_{t}^{\prime }/E=0.00009375$. In the test, exponential softening given in Equation (24) is taken into account. Parameters α and η are determined based on fracture energy $G_{f}=0.11$ N/mm. The residual stresses should asymptotically go to zero, so the first parameter α=1.0 and a complete loss of stiffness is accepted. The ductility parameter η is estimated as equal to 4000, which seems to be unrealistically huge for the DAP model, but it is connected with $G_{f}$ and related to the element size, so this value is truly correct. If modified von Mises definition in Equation (23) is employed, the ratio between compressive and tensile strength is equal to $k=f_{c}/f_{t}^{\prime }=12.7667$.

Plasticity with the HMH criterion for $F_{\mathrm{CDP}}^{p}$ is selected if the coupled model is turned on. The yield stress $\sigma_{y}$ is the uniaxial tensile strength, and $f_{t}^{\prime }=3$ MPa and isotropic linear hardening are applied. The hardening modulus h=0.5E is adopted. This value seems to be large, but it is known from [35] that, in the DAP model, hardening effects are connected with the fictitious (effective) configuration, i.e., with the material skeleton. The plastic part of the DAP model coupled with damage can also influence the development of microcracks. As shown by [55], the solution approaches the response as for pure damage when the value of h→∞. Two ways of coupling can be considered: total or elastic. For h=0.5E, the differences in results for manners of the coupling are clear enough.

As previously, the stress components versus strain $\epsilon_{11}$ are analyzed in the diagrams. The results for pure scalar damage model and the two different definitions of the equivalent strain measure are firstly compared. Figure 12a depicts the diagrams of stress components for the Mazars definition given in Equation (22), while Figure 12b illustrates the solution obtained for the modified von Mises measure defined in Equation (23). A more rapid decrease of stresses is noted for the second option. It is also observed that the maximum values of stresses $\sigma_{22}$ and $\sigma_{12}$ are about 50% smaller than for the Mazars definition. For both options, the uniaxial tensile strength $f_{t}^{\prime }=3$ MPa is kept and all stress components tend to zero, hence it can be concluded that, for pure scalar damage, Willam’s test is passed.

**Figure 13:**
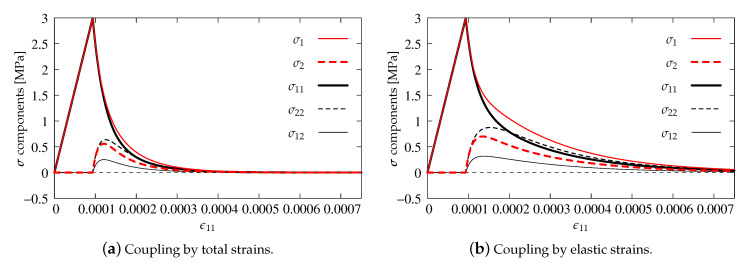
Comparison of stress components for DAP model—damage coupled to plasticity, modified von Mises definition.

The diagrams for two manners of coupling in the DAP model are presented in Figure 13. Now, only the modified von Mises definition is employed. The results have the same tendency as for pure scalar damage. The tensile strength is not exceeded and stresses tend to zero in the second phase of the test. However, after the peak, the descending paths run in such way that, for option $\tilde{\epsilon }\left(\epsilon \right)$ shown in Figure 13a, they are below those resulting from pure damage, cf. Figure 12b. This is the case of total coupling in the DAP model. The reverse is the case when the weak coupling $\tilde{\epsilon }\left(\epsilon^{e}\right)$ is considered, see Figure 13b. Now, all stress paths are above those presented for pure damage. It can generally be indicated that the DAP model passes Willam’s test in each case.

### 3.4. Isotropic Damage (IDA)

The data for the IDA model are the same as for pure damage in the previous section, but additional parameters P and R have to be determined. They decide how the damage parameter ω degrades bulk modulus *K* and shear modulus *G* in different ways. In Willam’s test, dilatancy only increases and θ≥0, so the constitutive relation given in Equation (37) is employed or, in other words, the damage reduction factor R=1.0, cf. also Equation (39). Other values of R are impossible in this test. The results for different R are shown by [51], where θ<0 is admitted e.g., for a splitting test. In this analysis, the value of the power P is introduced as smaller or larger than 1.0 to demonstrate deviations of the response of the IDA model. The power P=1.0 corresponds to the DAP model with pure damage.

**Figure 14:**
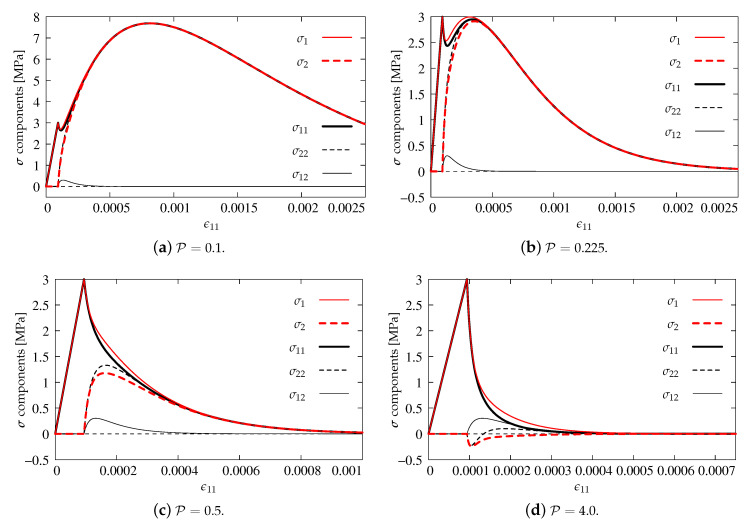
Comparison of stress components for the IDA model.

Again, in this subsection, axial strain–stress component’s diagrams are shown as previously. Figure 14a presents the results for P=0.1. The volumetric degradation is significantly reduced, so, after the first peak for $\sigma_{11}$ and $\sigma_{1}=f_{t}^{\prime }=3.0$ MPa and their quick and slight decrease, a second increase of stresses occurs up to value 7.69 MPa for $\epsilon_{11}=0.0008$. After passing this point, all diagrams descend, probably to zero. All components, apart from shear stress $\sigma_{12}$ which is zeroed, run together. An analogical behavior is observed for the next case depicted in Figure 14b. However, it is found that, for P=0.225, a second hump is reached when $\sigma_{1}$ equals 3.0 MPa, since the uniaxial tensile strength is not exceeded and the first condition of Willam’s test is passed. The second condition is also fulfilled, because the stresses approach zero. The results for cases P=0.5 and P=4.0 illustrated in Figure 14c,d satisfy Willam’s test as well. Increasing power P accelerates the process of stiffness degradation due to larger and larger reduction of bulk modulus *K*. In the case P=4.0, the steepest slope of the softening path for $\sigma_{11}$ and $\sigma_{1}$ is noticed. Negative values of $\sigma_{22}$ and $\sigma_{2}$ are manifested, but finally they return to zero. It means that the second condition is always passed, even if quite large values of P are introduced. It can therefore be concluded that the IDA model passes Willam’s test only if P≥0.225.

## 4. Discussion—Comparison of Models for Principal Stresses and Their Directions

In this section, selected eight cases are compared in the diagrams prepared to demonstrate a change of maximum and minimum principal stresses as well as of principal stress directions. These cases have previously been presented; they are characteristic options for all the models discussed in the paper. The comparison is done in order to show directly the different behavior of the selected concrete models for the same general data. The list of selected cases together with their most important features is given in Table 1. In the first column, corresponding acronyms of all cases investigated below are written. The second column includes full names of the models and their details. For additional help, in the last column, the number of figure related to the considered option is noted.

**Figure 15:**
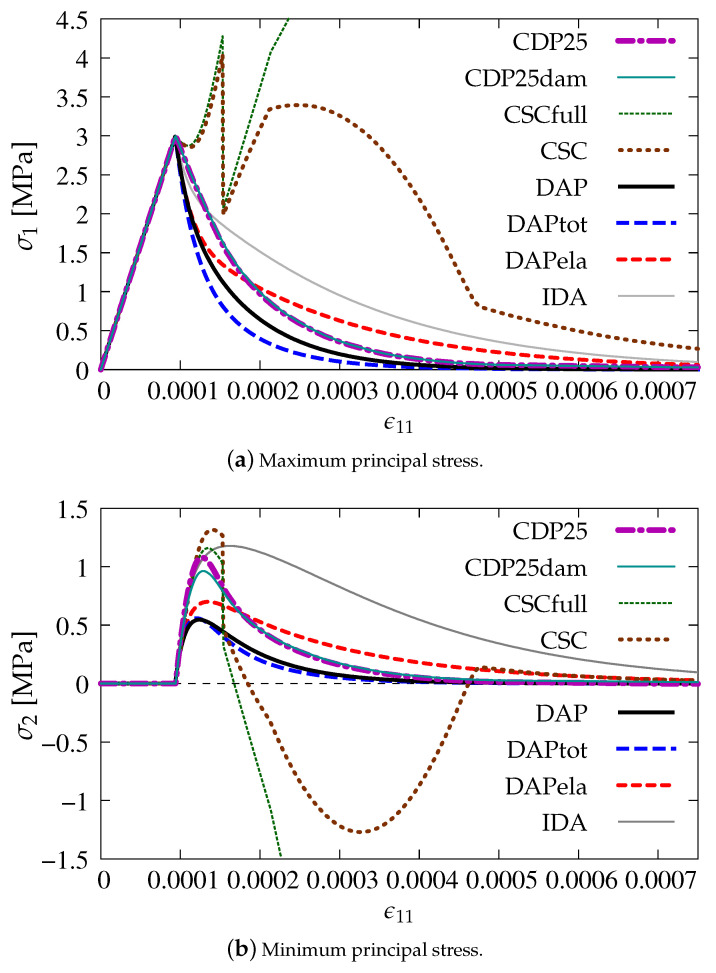
Comparison of models for evolution of principal stresses.

Figure 15a depicts the diagrams of maximum principal stress $\sigma_{1}$ as a function of axial strain $\epsilon_{11}$ (similarly to the presentation of results in Section 3) for the cases listed in Table 1. Figure 15b illustrates analogical results for the minimum principal stress $\sigma_{2}$. The diagrams for both options connected with the CDP model (CDP25—dark magenta dashed-dotted line and CDP25dam—dark green solid line) almost overlap for $\sigma_{1}$ and $\sigma_{2}$, but, for $\sigma_{2}$, the maximum value of stress is smaller for CDP25dam than for CDP25. It means that, for Willam’s test, where an active process is only considered without any type of unloading, the damage component in the CDP model does not matter. The most deviating diagrams are obtained for the CSC model, see the green dotted line for CSCfull and brown dotted line for CSC in Figure 15. Moreover, when $\sigma_{2}$ is taken into account, then negative values can appear. The results for the DAP model prove that coupling of the damage model with plasticity influences the response. The blue dashed curve (DAPtot) for full coupling by $\tilde{\epsilon }\left(\epsilon \right)$ in the DAP model is below the black solid curve (DAP) for pure damage, while the red dashed one (DAPela) for weak coupling by $\tilde{\epsilon }\left(\epsilon^{e}\right)$ is above the black one. A more ductile response is visible when only the elastic part of strains ***ϵ***^e^ stimulates the damage growth and, conversely, if the total strain tensor e influences in the damage process, then a more brittle response is noticed. However, apart from the results for the CSC model which are unacceptable, the diagram for the IDA model (gray solid line) gives the most ductile solution. It is observed for both principal stresses *σ*_1_ and *σ*_2_, cf. Figure 15a,b.

**Figure 16:**
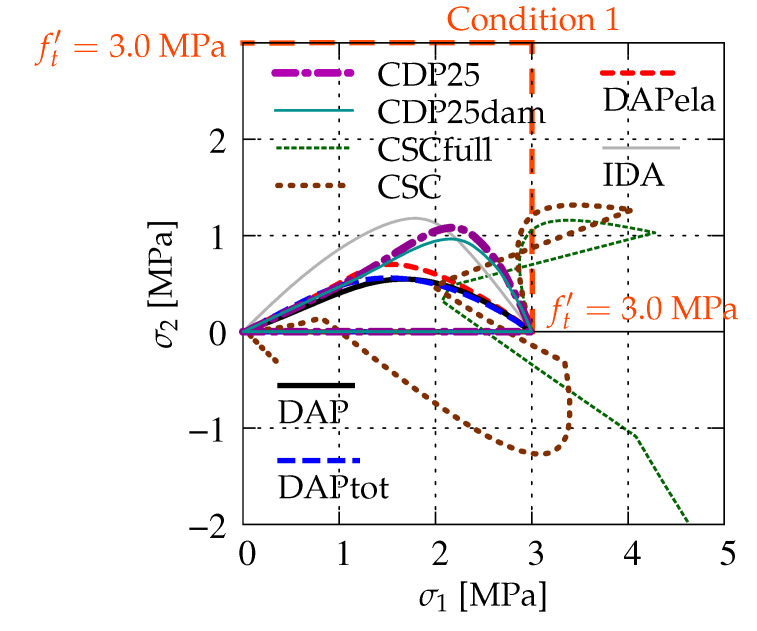
Comparison of models for evolution of principal stresses.

Figure 16 illustrates the evolution of principal stresses in first and fourth quadrants of the principal stress plane, cf. Figure 2 and Figure 5. It is seen that, in phase I, which corresponds to the uniaxial tension, $\sigma_{1}$ grows from zero to 3.0 MPa for all cases, while $\sigma_{2}$ is equal to zero in that stage. In phase II, when softening (cracking) occurs, the values of $\sigma_{1}$ decrease. At the same time, the values of $\sigma_{2}$ initially increase, but finally tend to zero. This is observed for all cases except CSCfull and CSC. The values of the principal stress $\sigma_{1}$ for the CSC model exceed the uniaxial strength $f_{t}^{\prime }$. After that, it seems that the stress paths tend to zero, but for about 2.0 MPa, this process is broken and they shoot up to infinity. For the case CSC, a second return is noticed, but near the origin this curve turns again and finally goes to infinity. The calculations for this case are interrupted. Hence, it is verified once more that the CSC model fails Willam’s test. The maximum values of $\sigma_{2}$ are obtained for IDA and next for CDP25. It can also be noticed that the differences between CDP25 and CDP25dam as well as DAP, DAPtot, and DAPela are small. The CDP, DAP, and IDA models behave in the principal stress space in a similar way.

**Figure 17:**
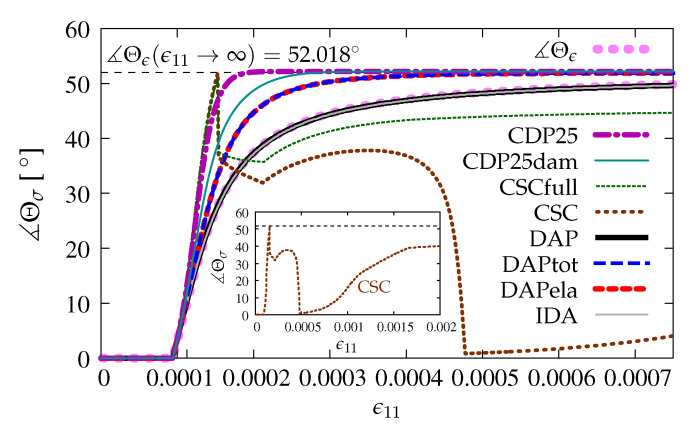
Comparison of models for principal directions.

The directions of principal stresses are not constant during the loading process, see also e.g., [2]. After the peak, at the beginning of phase II in Willam’s test, the rotation of principal directions increases fast, then this change slows down and finally tends to the angle with value $52.018^{\circ }$. The rotation of principal directions can be expressed by evolution of angle $\Theta_{\epsilon }$ for strains and $\Theta_{\sigma }$ for stresses during the loading process. In Figure 17 for two options DAP and IDA, where the scalar or isotropic damage is employed, the angle of principal directions $∡\Theta_{\sigma }$ for stresses and $∡\Theta_{\epsilon }$ for strains evolves in the same manner, since it is seen that, for pure damage (without any coupling), principal strains and stresses are coaxial. For the coupled version of the DAP model, no matter whether by total strains ϵ—case DAPtot or by elastic strains ***ϵ***^e^—case DAPela, the change of angle Θ_*σ*_ is faster than the change of Θ_*ϵ*_. Hence, the coaxiality of principal directions between strain and stress fields can be lost. It should also be noticed for options DAPtot and DAPela that both diagrams overlap and approach the final value 52.018° for the angle of principal directions. The existence of plasticity accelerates the effect of rotation of the principal stresses. It is confirmed for the CDP model as well. For option CDP25, the principal directions for stresses grow very fast and reach the angle with limit value 52.018° for *ϵ*_11_ = 0.0002. When the damage component is added in the CDP model, then, for option CDP25dam, this value of angle is achieved for *ϵ*_11_ = 0.0003. It seems that the value 52.018° is attained immediately for the CSC model, but next, for both cases CSCfull and CSC, the angle drops to about 35.0°, which corresponds to manifestation of the presence of the primary crack and then is slowly reduced till *ϵ*_11_ ≈ 0.00022. After that, the change of the angle depends on whether stronger or weaker shear retention is assumed in the CSC model. For option CSCfull, the value of the angle slowly increases up to about 45.0°. When option CSC is considered, the angle decreases almost to zero and afterwards increases to 40.0° for *ϵ*_11_ = 0.002, see the internal subfigure in Figure 17.

## 5. Conclusions

In the paper, popular concrete models are selected to verify if they pass or fail Willam’s test [1]. This test is a one finite element benchmark containing two phases: uniaxial tension till the tensile strength is achieved and softening in the biaxial tension–shear regime. It is proved that the results of the test for each concrete model can be different even if the parameters are calibrated in such a manner that the models exhibit almost identical behavior in uniaxial tension.

The following models are tested: concrete damaged plasticity (CDP) and concrete smeared cracking (CSC) models delivered in the ABAQUS software [4], the da-mage-plasticity (DAP) model without or with coupling of both theories by elastic or total strain tensor and finally an isotropic upgrade (IDA) of the damage model where the volumetric-deviatoric split is applied. The DAP and IDA models are implemented in the FEAP package [5].

Table 2 summarizes the content of the paper. The CDP model passes Willam’s test, but only when the dilatancy angle ψ is smaller than $35^{\circ }$; otherwise, exaggerated dilatancy is observed. Please note that a large dilatancy angle, i.e., $\psi \geq 49^{\circ }$, is considered in several works, cf. [21,54,56,57]. The recommendation to use the model for $\psi \leq ∼\;35^{\circ }$ is similar to that given in [22], where punching shear in slabs is simulated. The CSC model fails Willam’s test, even if the effect of shear retention is substantially suppressed. On the other hand, the DAP model passes the test independently of the presence and kind of coupling with plasticity. When the scalar damage is upgraded to the isotropic version as in [48] or in the fashion of the IDA model, then the parameters can decide about passing or failing Willam’s test. Here, for the IDA model, the power P governs the degradation of the volumetric part of the stiffness, but it should be equal to or larger than 0.225 to satisfy the first condition and thus pass Willam’s test.

As stated in the Introduction, failing Willam’s test by a given concrete model raises serious doubts concerning its ability to describe properly the structural behavior with predominant mixed-mode fracture, e.g., RC beams failing in shear as shown in [12]. Therefore, among the investigated models, the CSC model cannot be recommended for such structural analyses. The other models, i.e., CDP, DAP, and IDA, seem to be well suited for nonlinear FE computations of concrete structures with the predominant mixed-mode fracture; however, the restrictions mentioned above for the CDP and IDA models should be taken into account. As a general suggestion—in the authors’ opinion, a verification using Willam’s test should be mandatory when any new material model for concrete is proposed.

## Figures and Tables

**Table 1:** Models considered in comparison.

Acronym	Model	Crucial details	Figure
CDP25	concrete damaged plasticity	dilatancy angle $\psi =25^{\circ }$	Figure 9b
CDP25dam	concrete damaged plasticity	dilatancy angle $\psi =25^{\circ }$, tensile damage—Figure 8a	Figure 10
CSCfull	concrete smeared cracking	shear retention: $\rho_{\mathrm{cl}}=1.0$, $\;\epsilon_{\max}=10.0$	Figure 11a
CSC	concrete smeared cracking	shear retention: $\rho_{\mathrm{cl}}=1.0$, $\;\epsilon_{\max}=0.001$	Figure 11b
DAP	damage	modified von Mises definition	Figure 12b
DAPtot	damage-plasticity	coupling by total strains $\tilde{\epsilon }\left(\epsilon \right)$	Figure 13a
DAPela	damage-plasticity	coupling by elastic strains $\tilde{\epsilon }\left(\epsilon \right)$	Figure 13b
IDA	isotropic damage	power P=0.5	Figure 14c

**Table 2:** Usage range of Willam’s test.

Model	Condition		Final	Restriction
Acronym	1	2	Assessment	of Usage
CDP	+	+/−	conditionally	$\psi \leq \;∼\;35^{\circ }$
CSC	+/−	−	no	
DAP	+	+	yes	
IDA	+/−	+	conditionally	P≥0.225

## Abbreviations

The following abbreviations are used in this manuscript:

CDP	concrete damaged plasticity model
CSC	concrete smeared cracking model
DAP	damage-plasticity model
FE	finite element
FEM	finite element method
IDA	isotropic damage model
MCFT	modified compression field theory
RC	reinforced concrete
